# Exploring Bioactive Compounds in Brown Seaweeds Using Subcritical Water: A Comprehensive Analysis

**DOI:** 10.3390/md21060328

**Published:** 2023-05-26

**Authors:** Jin-Seok Park, Ji-Min Han, Yu-Na Shin, Ye-Seul Park, Ye-Ryeon Shin, Sin-Won Park, Vikash Chandra Roy, Hee-Jeong Lee, Yuya Kumagai, Hideki Kishimura, Byung-Soo Chun

**Affiliations:** 1 Department of Food Science and Technology, Pukyong National University, 45 Yongso-ro Namgu, Busan 48513, Republic of Korea; jin1931@pukyong.ac.kr (J.-S.P.); wlals383@gmail.com (J.-M.H.); bellyshin@naver.com (Y.-N.S.); tmf0212@gmail.com (Y.-S.P.); tlsdpfus12@pukyong.ac.kr (Y.-R.S.); psw3475@gmail.com (S.-W.P.); 2 Institute of Food Science, Pukyong National University, 45 Yongso-ro Namgu, Busan 48513, Republic of Korea; vikashft@hstu.ac.bd; 3 Department of Fisheries Technology, Hajee Mohammad Danesh Science and Technology University, Dinajpur 5200, Bangladesh; 4 Department of Food Science and Nutrition, Kyungsung University, Busan 48434, Republic of Korea; 5 Laboratory of Marine Chemical Resource Development, Faculty of Fisheries Sciences, Hokkaido University, Hakodate 041-8611, Japani-dulse@fish.hokudai.ac.jp (H.K.)

**Keywords:** brown seaweed, subcritical water extraction, bioactive compounds, biological activity

## Abstract

In this study, we characterized the bioactive properties of three important brown seaweed species, *Sargassum thunbergii*, *Undaria pinnatifida*, and *Saccharina japonica*, by subcritical water extraction (SWE), as these species are well known for their beneficial health effects. Their physiochemical properties, including potential antioxidant, antihypertensive, and α-glucosidase inhibitory activity, and the antibacterial activity of the hydroysates were also analyzed. The highest total phlorotannin, total sugar content, and reducing sugar content in the *S. thunbergii* hydrolysates were 38.82 ± 0.17 mg PGE/g, 116.66 ± 0.19 mg glucose/g dry sample, and 53.27 ± 1.57 mg glucose/g dry sample, respectively. The highest ABTS^+^ and DPPH antioxidant activities were obtained in the *S. japonica* hydrolysates (124.77 ± 2.47 and 46.35 ± 0.01 mg Trolox equivalent/g, respectively) and the highest FRAP activity was obtained in the *S. thunbergii* hydrolysates (34.47 ± 0.49 mg Trolox equivalent/g seaweed). In addition, the seaweed extracts showed antihypertensive (≤59.77 ± 0.14%) and α-glucosidase inhibitory activity (≤68.05 ± 1.15%), as well as activity against foodborne pathogens. The present findings provide evidence of the biological activity of brown seaweed extracts for potential application in the food, pharmaceutical, and cosmetic sectors.

## 1. Introduction

Seaweed, multicellular marine organisms capable of rapid growth due to their high photosynthetic rates, are the largest source of biomass within the marine environment [1]. Not only are they a rich source of nutrients, such as carbohydrates, vitamins, and minerals, but they also have bioactive compounds such as phenols, fatty acids, and pigments, which have recently received a lot of attention for their antioxidant, antidiabetic, antihypertensive, and anticancer properties [2]. Seaweed are classified into three groups based on their pigments: red (Rhodophyta), brown (Phaeophyta), and green (Chlorophyta) [3]. Among them, brown seaweed is the most consumed in the world, accounting for about 66.5% of total production [4]. In addition, brown seaweed contains high amounts of unique bioactive compounds such as fucoidan and fucoxanthin, reported to have higher antioxidant and antibacterial activity [5]. Recently, the discovery of biologically active substances from natural sources has gained significant attention due to its potential impact. Marine organisms are a rich source of various bioactive compounds, including terpenoids, polyethers, polyketides, polyphenolic compounds, lipoproteins, and peptides, as well as proteins, glycoproteins, and polysaccharides that differ from those found in land-based organisms [6].

Seaweed produce physiologically active metabolites needed to thrive in a highly competitive environment [2]. These secondary metabolites have complex chemical structures and exhibit antioxidant, antimicrobial [7], anti-photoaging [8], anticancer [9], anti-inflammatory [10], antitumor [11], and antidiabetic [12] activities. In addition, due to their natural origin, these compounds have garnered attention and demonstrate potential applicability in various domains, including dietary supplements and cosmetics [13]. Recent studies have revealed that the bioactive compounds in seaweed can enhance the stability of oxidation-sensitive food products [14]. However, several studies have also mentioned that green extraction methodologies such as subcritical water extraction (SWE) can maximize the valorization of seaweed biomass targeting to obtain the valuable components without any chemical residue [15,16,17]. 

Extraction is the most critical operation for obtaining bioactive compounds, and the extraction techniques, operating parameters, and pretreatment methods are crucial in determining extract quality and yield [18]. Organic solvents, such as ethanol, methanol, and *n*-hexane or hydrothermal extraction are commonly used for natural product extraction. However, the organic solvent concentration affects the content of active ingredients, with higher concentrations decreasing extraction yields and increasing manufacturing costs and safety risks due to solvent residue [19]. Although hydrothermal extraction is safe and yields a higher extraction efficiency than organic solvents, it has limitations such as decreased productivity and lower content of active ingredients due to high temperatures and prolonged extraction times. To address these issues, various green extraction technologies have been developed [20]. Subcritical water extraction (SWE) is a typical green extraction technology at the critical temperature (374 °C) under critical pressure (1~22.1 MPa) of pure water. The dielectric constant (ε), the main property that describes the structure of subcritical water, is 80 at room temperature (25 °C, 0.1 MPa) and 25 when the temperature and pressure are increased to 250 °C, 2.5 MPa, which is similar to the dielectric constants of methanol (ε = 32.6), ethanol (ε = 24.3), and acetone (ε = 20.7). Under these conditions, the polarity of the solvent is widely controlled by controlling the temperature and pressure of water, enabling selective extraction of bioactive substances [21].

Although several studies have been conducted to extract high-value compounds from several algal biomasses using SWE, the bioactivity such as antihypertensive activity, α-glucosidase inhibitory activity, and other functionality are still to be explored. Hence, in this study, we utilized SWE to prepare bioactive extracts of three types of mass-produced brown seaweed (*Sargassum thunbergii*, *Undaria pinnatifida*, and *Saccharina japonica*) from the coastal waters of South Korea to identify the bioactive compounds that have commercial value and explore their potential for the development of food, cosmetics, and pharmacologically active products. We also investigated the functional components (polysaccharides and phenolic compounds) in the extracts and analyzed and compared their biological properties, including antioxidant, antibacterial, antihypertensive, and antidiabetic effects. 

## 2. Results and Discussion

### 2.1. Proximate Composition

The results of the analysis of the proximate composition of the three dried brown seaweed species are presented in Table S1. They were mostly composed of carbohydrates (49.58 ± 0.02% for *S. thunbergii*, 54.57 ± 0.12% for *U. pinnatifida*, and 62.87 ± 0.94% for *S. japonica*). The high carbohydrate content of brown seaweed suggests that it could be an important source of polysaccharides for industrial use. In particular, polysaccharides such as fucoidans, alginates, and laminarin are only found in brown seaweed [22]. Moreover, polysaccharides such as fucoidan, alginate, and laminarin have been reported to possess antitumor, antimicrobial, immunostimulatory, and anti-inflammatory activities, having potential applications in drug delivery, tissue engineering, and skin repair [23]. Additionally, the investigated seaweed had high ash (mineral) content ranging from 16.79 ± 0.98% to 26.02 ± 0.34%, which can be attributed to the mineral absorption through the entire surface of the talus, rather than through roots, similar to other marine organisms [24].

### 2.2. Extraction Efficiency

The extraction efficiencies varied from 68.40 ± 0.44% to 81.88 ± 1.35% in the different types of seaweed (Table 1). *U. pinnatifida* had the highest extraction efficiency while *S. thunbergii* showed the lowest. The constitutional differences in the raw materials are possibly the reason behind the differences in extraction efficiency [25]. Moreover, the different extraction yields obtained under similar conditions can be due to differences in the content of polysaccharides, proteins, ash, and other compounds in seaweed. Further research is needed to better understand these findings.

### 2.3. Color and Maillard Reaction Products (MRPs)

The color analysis of brown seaweed subcritical water extracts (BSEs) is shown in Table 1 and actual images are shown in Figure S1. The L* value representing the brightness value varied from 30.33 ± 2.34 to 34.42 ± 0.89; slight differences were observed depending on the algae, but not all differences were significant. The a* value (red to green) ranged from 1.63 ± 0.10 to 4.53 ± 0.38, and the b* value (yellow to blue) ranged from 0.48 ± 0.22 to 2.92 ± 0.33, indicating that *U. pinnatifida* has higher redness than other seaweed extracts. *U. pinnatifida* has been shown to have a higher amount of β-carotene and carotenoids than other types of brown seaweed [26]. The extract’s lightness depends on the pigments and hygroscopic substances contained in the extract, while the darkness depends on the depolymerization of polymeric materials and the formation of MRPs [27].

The carbonyl group of reducing sugars and the free amino group react during the subcritical water reaction of seaweed to form MRPs, browning compounds [28]. The MRP content was analyzed through the absorbance at 294 nm and 420 nm. The first can be used to quantify the intermediate compounds during MRP formation while the second determines the browning intensity caused by different polymeric substances such as melandion. The ratio of these two absorbances (A294/A420) measures the efficacy of UV-absorption and conversion of UV-absorption substances into different polymers for browning intensity [29]. The mean UV absorbance, browning intensity, and absorbance ratio ranged from 2.163 ± 0.028 to 2.980 ± 0.029, 0.188 ± 0.003 to 0.306 ± 0.001, and 9.328 ± 0.029 to 11.506 ± 0.155, respectively. The production of MRPs from hydrolysates of algae has been reported to have several biological activities, including antioxidant activity, metal chelating properties, antimicrobial activity, and antihypertensive activity [27]. 

### 2.4. Total Phlorotannin Content (TPC), Total Sugar Content (TSC), and Reducing Sugar Content (RSC)

The TPC, TSC, and RSC of the BSEs are shown in Table 2. Previous studies on seaweed have shown that the highest yields of phlorotannin compounds are extracted between 170–210 °C [30,31,32]. Phlorotannins are the major polyphenolic compounds in brown seaweed, and the TPC content was 31.32 ± 0.50, 37.94 ± 0.30, and 38.82 ± 0.17 mg PGE/g in *U. pinnatifida*, *S. japonica*, and *S. thunbergii*, respectively. Seaweed produce physiologically active substances, such as phenolic compounds, which can help them to survive under high internal pressure, high salt concentrations, and low temperatures and provides protection against pathogens [3]. In addition, the phenolic compounds in brown seaweed have numerous biological activities, such as antitumor, anticancer, antimicrobial, antiviral, anti-obesity, antiproliferative, anti-inflammatory, antidiabetic, and antioxidant properties [33]. The production of phenolic compounds in seaweed depends on various factors, such as the species, region, and environmental conditions, which can affect the quantity and types of phenolic compounds produced [34].

Brown seaweed consists of various polysaccharides such as fucoidan, alginate, and laminarin, which depend on the monosaccharides in seaweed. Fucoidans, in particular, are heat-unstable polysaccharides [35], which may decompose due to high temperature or through a Maillard reaction with other compounds [36]. Seaweed such as *U. pinnatifida* and *S. japonica* contain high levels of fucoidans. When extracted using subcritical water at 180 °C, the TSC of *U. pinnatifida* was 23.88 ± 0.42 mg glucose/g dry sample and that of *S. japonica* was 33.27 ± 0.51 mg glucose/g dry sample, which was lower than in the other seaweed.

The production of biofuels or other valuable products from seaweed polysaccharides requires conversion into fermentable reducing sugars, and subcritical water hydrolysis is considered a promising technology for this purpose. This method has been successfully used to produce reducing sugars from woody biomass such as cellulose and hemicellulose as well as from algae-derived biomass [37]. Among the three species studied, *S. thunbergii* produced the highest amount of reducing sugars after subcritical water treatment, with 53.27 ± 1.57 mg glucose/g dry sample, followed by *S. japonica* with 46.00 ± 1.82 mg glucose/g dry sample.

The correlation between MRPs and sugar contents is crucial to understanding the browning intensity and flavor development in various food systems. In our study, we explored the relationship between the a294/a420 ratio, a measure of UV absorption efficacy and browning intensity, and the total and reducing sugar contents (Table S2). Our findings revealed a weak negative correlation between the a294/a420 ratio and total sugar content. This suggests that the total sugar content tends to decrease as the browning intensity increases. Furthermore, our study also unveiled a strong negative correlation between the a294/a420 ratio and reducing sugar content. Reducing sugars play a crucial role in the Maillard reaction, acting as precursors for the formation of Maillard reaction products [38]. The negative correlation observed implies that as the browning intensity increases, the availability of reducing sugars for the Maillard reaction decreases. This finding contributes to our understanding of the dynamics between reducing sugars and the formation of Maillard reaction products.

### 2.5. Monosaccharide Composition and Molecular Weight Analysis

The polysaccharides present in brown seaweed are the primary source of fucoidan, a combination of sugars such as fucose, galactose, xylose, and mannose [5]. These polysaccharides have a diverse range of pharmacological activities, including anti-oxidative agents. After analyzing the monosaccharide composition of BSEs, the composition varied slightly depending on the seaweed. However, the primary components were fucose, galactose, glucose, xylose, and mannose (Table 3, Figure S2). Among these, the lowest fucose percentage was observed in *U. pinnatifida* (19.55%), whereas *S. thunbergii* had the highest (36.71%). *S. thunbergii* had the lowest galactose percentage of (21.49%), whereas *U. pinnatifida* had the highest (48.84%). For glucose, *S. japonica* had the lowest percentage (14.39%), whereas *S. thunbergii* had the highest (22.96%). For xylose, the lowest percentage was found in *U. pinnatifida* (4.02%), and the highest was observed in *S. thunbergii* (11.37%). In the case of mannose, *S. japonica* had the lowest percentage (4.73%), whereas *U. pinnatifida* had the highest (9.61%). These findings are consistent with previous studies that have reported fucose, galactose, and glucose as major components and xylose, mannose, and rhamnose as minor ones [39]. However, the proportions may differ due to the species, environmental conditions, geography, harvesting timing and extraction, and differences in separation methods [40]. Moreover, the physiological activity of polysaccharides may vary depending on the content and ratio of monosaccharides [41]; particularly, the fucose to xylose ratio can provide information about the biological activity of BSEs [42]. 

The molecular weight of the BSEs was determined using gel permeation chromatography (GPC); Table 3, Figure S3). The number molar mass (Mn), average molar mass (Mw), and polydispersity index (PI, Mw/Mn) were in the range of 331–498 Da for Mn, 982–1514 Da for Mw, and 2.58–3.04 for PI, respectively. These values showed slight variation among the seaweed species. The PI values of the extracts obtained by subcritical water extraction indicate non-uniform compounds in the final products. This study reveals the efficacy of subcritical water for extracting polysaccharides of different molecular weights from different types of seaweed. The reactor temperature and time are the major concerns for varying the molecular weight of polysaccharides treated by SWE. Among them, the temperature in SWE significantly affects the molecular weight’s magnitude [43]. Low molecular size polysaccharides can be used for the cross-linking properties of alginates [44] and the conversion of algae-derived biomass [45] to enhance their functional properties. Ali et al. revealed that a low molecular weight polysaccharide showed higher antioxidant, antimicrobial, and anticancer activity over a high molecular weight polysaccharide [46]. This study reveals that BSEs contain comparatively lower molecular sizes of polysaccharides that have the potential for commercial application in various industries.

### 2.6. Antioxidant Activity

Seaweed extracts and their fractions are a rich source of antioxidants, with various types of seaweed yielding different kinds of antioxidants [47]. Polyphenols are known for their multifunctional antioxidant activity due to their phenolic ring, which acts as an electron trap, scavenging peroxide, superoxide-anion, and hydroxyl radicals [48]. To assess the antioxidant activity of the BSEs, we measured 2,2′-azino-bis (3-ethylbenzothiazoline-6-sulphonic acid) (ABTS) and 2,2-diphenyl-1-picrylhydrazyl (DPPH) and used the reducing power (FRAP) assay, as shown in Table 4. The BSE with the highest antioxidant activity was *S. japonica*, with 124.77 ± 2.47 mg ABTS^+^, 45.56 ± 0.23 DPPH, and 34.47 ± 0.49 FRAP Trolox equivalent (TE)/g of dry sample. Meanwhile, the BSE with the lowest antioxidant activity was *U. pinnatifida*, with ABTS, DPPH, and FRAP of 79.13 ± 1.78 mg TE/g of dry sample, 35.29 ± 0.24 mg TE/g of dry sample, and 27.02 ± 0.56 mg TE/g of dry sample, respectively. The difference in the antioxidant activity among the brown seaweed is mainly attributed to the content of phenol compounds [49]. In addition, the correlation results between TPC and ABTS^+^, DPPH, and FRAP were *R*^2^ = 0.729, 0.888, and 0.873, respectively (Table 5), indicating a moderate correlation between phenolic compounds and antioxidant activity. Previous studies have shown a strong correlation between TPC and antioxidant activity [21], further confirming that SWE extracts from brown seaweed are an excellent source of natural antioxidants.

### 2.7. Antihypertensive Activity

Recently, hypertension has been causing various public health problems as a result of modern lifestyles and is one of the major risk factors contributing to the development of cardiovascular diseases [50]. Inhibition of angiotensin-I converting enzyme (ACE), a key regulator of the renin-angiotensin system, could be a viable approach to lower blood pressure [51]. Despite the strong effects of synthetic ACE inhibitors, long-term administration can lead to undesirable side effects such as coughing, allergic reactions, skin rashes, taste disorders, kidney damage, and angioneurotic edema [52]. Therefore, active substances from natural sources could be used as ACE inhibitors with fewer side effects. We found that BSEs have the potential for antihypertensive activity showing efficacy in a range of 56.55% ± 0.26% to 59.77% ± 0.14%. The antihypertensive activity of any extract largely depends on its bioactive compounds. All extracts had high TPC content; thus, their antioxidant activities might explain their antihypertensive activities. When using captopril (0.1%), a synthetic ACE inhibitor used in the treatment of hypertension and congestive heart failure, as a reference material, we obtained an inhibition rate of 99.60% ± 0.28%. The BSEs have sufficient activity to be used as a natural ACE inhibitor, with the potential for reduced side effects compared to synthetic ACE inhibitors. 

### 2.8. α-Glucosidase Inhibitory Activity

According to the International Diabetes Federation, the number of people with diabetes worldwide was estimated to be 463 million in 2019, which is expected to increase to 578 million by 2030 and 700 million by 2045 [53]. α-glucosidase is responsible for breaking down oligosaccharides into monosaccharides, increasing blood sugar levels; thus, inhibiting its activity could be a promising treatment for diabetes. While synthetic antihyperglycemic drugs such as acarbose, voglibose, or insulin can alleviate diabetes, they often have side effects or require injections [54]. Therefore, natural preparations could be an alternative as blood-glucose-lowering agents. In the present study, we determined the α-glucosidase inhibitory activity of BSEs and found that *S. thunbergii* showed the highest enzyme inhibition rate of 79.26% ± 0.19%, followed by *U. pinnatifida* with 38.52% ± 0.80%. This suggests that BSEs could be a potential natural blood-sugar-lowering agent. However, further research is needed to understand their mechanism of action and specific effects on blood sugar levels. The correlation between the bioactive compounds present in BSEs and α-glucosidase inhibitory activity is shown in Table 5. The correlation results show a positive coefficient, indicating that the BSEs have the potential to reduce the diabetic tendency of human beings. Polyphenols from brown seaweed, especially phlorotannins, are effective at reducing the impact of metabolic disorders and potent for anti-diabetic activity [55]. The low molecular weight seaweed oligosaccharides have already been proven as an effective bio-component for reducing obesity and has antidiabetic effects [56]. These results show that the BSEs obtained by subcritical water hydrolysis are an excellent source of polyphenolic compounds and oligosaccharides. The presence of these bio-components results in higher antioxidant activity and antidiabetic activity, thus showing the potential of brown seaweed for commercial applications.

**Table 5:** Pearson’s correlation coefficients between chemical properties and biological activities.

Trait	TPC	TSC	RSC	ABTS^+^	DPPH	FRAP	α-Glucosidase Inhibitory
TPC	1	0.659 ^ns^	0.888 **	0.841 **	0.980 **	0.994 **	0.473 ^ns^
TSC	-	1	0.897 **	0.238 ^ns^	0.524 ^ns^	0.623 ^ns^	0.974 **
RSC	-		1	0.560 ^ns^	0.810 **	0.878 **	0.788 *
ABTS^+^				1	0.912 **	0.854 **	0.017 ^ns^
DPPH					1	0.983 **	0.321 ^ns^
FRAP						1	0.436 ^ns^
α-glucosidase inhibitory							1

** The correlation is significant at the 0.01 level. * The correlation is significant at the 0.05 level. ^ns^: not significant.

### 2.9. Antibacterial Activity

Using the well diffusion method, the antimicrobial activity of the BSEs was evaluated against representative foodborne pathogens, including Gram-positive bacteria (*B. cereus* and *S. aureus*) and Gram-negative bacteria (*E. coli* and *S. enterica*). We found that the BSEs showed greater activity against Gram-positive bacteria, with *S. thunbergii* exhibiting the highest antimicrobial activity, showing an inhibition zone of 18 mm against *B. cereus* and 22 mm against *S. aureus* at the highest concentration of 50 mg/mL. *S. japonica* followed *S. thunbergii*, exhibiting an inhibition zone of 14 mm against *B. cereus*, and *U. pinnatifida* exhibited an inhibition zone of 15 mm against *S. aureus*. However, except for *S. thunbergii* (*E. coli* 14 mm, *S. enterica* 14 mm), none of the extracts exhibited antibacterial activity against both Gram-negative bacteria, even at the highest concentration tested (Table 6). This result is consistent with previous studies that confirmed the antibacterial effect of various extracts against Gram-positive bacteria [57]. This phenomenon is believed to result from the outer membrane surrounding the cell wall of Gram-negative bacteria, which can limit extract penetration into cells, thereby reducing their antibacterial activity [58]. Additionally, resistance to antimicrobials can arise from the presence of hydrophobic lipopolysaccharides and hydrophilic porins [59]. Mutations in the hydrophilic porin structure or changes in the hydrophobicity of the outer membrane of Gram-negative bacteria can increase resistance. Gram-positive bacteria lack this unique outer membrane and are more sensitive to drug molecules [60]. The mechanisms by which extracts of natural products inhibit microorganisms are complex and may involve synergistic or antagonistic effects between compounds or the specificity of a compound to a particular microorganism since both major and minor compounds can contribute to antimicrobial activity. Therefore, it is difficult to predict the exact individual compounds that inhibit microorganisms [61].

## 3. Materials and Methods

### 3.1. Materials 

Among the brown seaweed used in this experiment, the brown seaweed samples were purchased from different places. Among them, *S. thunbergii* was collected from Jeju Island (PARAJEJU, Jeju-do, Republic of Korea), and *U. pinnatifida* and *S. japonica*, cultivated in the Gijang area of Busan, were purchased from Dndn-Bada (Gijang-gun, Republic of Korea). All of the brown seaweed samples were cleaned with fresh water to remove non-target materials and to attach salts and minerals. Then, the samples were dried at 40 °C for 72 h. The dried samples were processed using a PN SMKA-4000 mixer (PN Poong Nyun Co., Ltd., Gyeonggi-do, Republic of Korea) and subsequently sieved through a 710 μm mesh to ensure a uniform particle size. The seaweed powders were then stored at −40 °C in a sealed container for future use. 99.99% pure nitrogen gas was obtained from Busan, Republic of Korea, to obtain the target pressure in the subcritical water. The standards were purchased from Sigma-Aldrich and the chemicals and reagents were obtained from Samchun Pure Chemical Co., Ltd. Gyeonggi, Republic of Korea.

### 3.2. Proximate Composition Analysis

The proximate composition of the dried brown seaweed powder was analyzed following the Association of Official Agricultural Chemists methods as previously described [7].

### 3.3. Subcritical Water Extraction 

A batch-type stainless-steel-made reactor (Phosentech, Daejeon, Republic of Korea) was used for the SWE of the brown seaweed as previously reported [21] with minor modifications. In the 500 cm^3^ reactor, 15 g of the seaweed samples was mixed with double distilled water at a ratio of 1:20 (*w/v*). The pressure was maintained constant (3 MPa) using nitrogen gas and the extraction time and temperature were 30 min and 180 °C in all extractions, respectively. The solid–solvent mixtures were stirred at 200 rpm using a double four-blade impeller during extraction. As for the heating time, for *S. thunbergii*, *U. pinnatifida*, and *S. japonica*, we observed average heating times of 24 min, 26 min, and 27 min, respectively.

The solid–solvent mixture was immediately cooled using the chilled water line attached to the reactor. The BSEs were filtered using filter paper (CHMLAB GROUP, F1091-110) and stored at 4 °C for further experiments. The extraction efficiency was calculated based on the weight of the solid residue after drying at 55 °C for 48 h to ensure complete moisture removal, resulting in a completely dewatered solid residue, using the formula:

Extraction efficiency (%) = (W − W1/W) × 100 (1)

where W stands for the original sample taken for hydrolysis and W1 is the weight of the dried solid extract after hydrolysis.

### 3.4. Color

To determine the BSEs’ color, their different parameters (L*, a*, and b* values) were measured by a colorimeter (Lovibond RT series, The Tintometer Ltd., Amesbury, UK). The values of L* can vary from 0–100, indicating the lightness of the extracts, where a* stands for red to green and b* stands for yellow to blue. The standard plate values used were L* = 94.92, a* = −1.04, and b* = 0.19. Each measurement was conducted three times and the average was calculated.

### 3.5. MRP

The UV absorbance and browning of the MRP samples were measured according to previously described methods [32]. Each hydrolysate was diluted 20× with distilled water and the absorbance was measured at 294 and 420 nm using a Synergy HT microplate reader (BioTek Instruments, Winooski, VT, USA). The absorbance ratio (A294/A420) was calculated to monitor the transformation of the UV-absorbing compound into brown polymers.

### 3.6. TPC

The TPC content of the BSEs was determined using a previously published method with slight modifications [7]. The characteristic color can be obtained in the analysis in the presence of Folin–Ciocalteu (FC) reagent and the polyphenolic compounds of the extracts. To measure TPC, 0.3 mL of the extract was mixed with 0.1 mL of 10% FC phenol reagent and allowed to stand for 5 min. Then, 2.0 mL of 10% Na_2_CO_3_ was added to the mixture, which was vortexed for 30 s and kept in the dark for 90 min. The absorbance of the resulting solution was measured at 765 nm, and TPC was expressed as mg phloroglucinol equivalent per gram of dry sample (mg PGE/g of dry sample), with phloroglucinol used as the standard reagent for comparison.

### 3.7. Total Sugar and RSC

We followed our previous method to determine the total sugars of the BSEs [7]. Briefly, a 3 mL solution of extract and sulfuric acid was prepared at a 1:3 mixing ratio; afterwards, a small amount (0.45 mL) of 40% phenol solution was also added to the mixture. The prepared solution was then incubated for 5 min in a hot water bath. After incubation, the reactant mixture was cooled at room temperature and a microplate reader measured the absorbance at 490 nm. A standard curve of glucose was prepared, and the glucose content of the extracts was quantified using the standard equation from the curve. The obtained results were also expressed as mg glucose/g of dry sample.

The 3,5-dinitrosalicylic (DNS) colorimetric assay, as described by Ali [46], was used to measure the RSC of the extracts. The DNS solution was prepared by mixing DNS (1 g) and a sodium–potassium tartare–sodium hydroxide solution (30 g/80 mL, *w*/*v*), adjusting the final volume to 100 mL with double distilled water. Next, 1 mL of the extract was combined with 4 mL of DNS solution, incubated in boiling water for 5 min and cooled to room temperature. The sample absorbance was measured at 540 nm using a microplate reader, and the values were compared to glucose as a standard. The RSC values were expressed as mg glucose/g dry sample.

### 3.8. Monosaccharide Analysis

The monosaccharide composition of the BSEs was analyzed using an Ion Chromatograph ICS-5000 with pulsed amperometric detection (Dionex, Sunnyvale, CA, USA) equipped with a CarboPac_SA10G column (4 × 50 mm, Dionex, Sunnyvale, CA, USA). To prepare the sample solution, 50 mg of lyophilized BSE powder was mixed with 5 mL of 12M sulfuric acid and 25 mL water and hydrolyzed at 120 °C for 2 h to convert it into monosaccharides. The resulting hydrolysate was filtered using a 0.2 μm syringe filter and used as the sample solution. The following HPLC conditions were used: solvent A, DIW; solvent B, NaOH 100 mM; 0–24 min (6% B), 24–25 min (6–100% B), 25–30 min (100% B), 30–31 min (100–6% B), 31–50 min (6% B); flow rate, 0.6 mL/min; injection volume, 10 μL.

### 3.9. Molecular Weight Analysis

The molecular weight of the BSEs was determined using GPC (Thermo Dionex HPLC Ultimate3000 RI System, Thermo, Waltham, MA, USA). First, the dried samples were dissolved in deionized water at a concentration of 10 mg mL^−1^. The solution was then filtered through a 0.45 µm pore size PTFE-H syringe filter and degassed. The GPC program was set with an injection volume of 10 µL, and a developing solvent of 0.1 M NaN_3_ in water was used with a flow rate of 1 mL min^−1^. Water Ultrahydrogel Columns 120, 500, and 1000 connected in series were used. The molecular weight was determined based on the calibration curve of pullulan standards (3.42 × 10^2^ to 8.05 × 10^5^ g mol^−1^). The results were analyzed using Chromeleon 6.8 Extension-pack software (Thermo, Waltham, MA, USA).

### 3.10. Biological Activity

#### 3.10.1. Antioxidant Activity (DPPH, ABTS, and FRAP Assay)

The radical scavenging activity of the BSEs was measured using DPPH, ABTS, and FRAP assays, as previously described [7], with minor modifications. The absorbance of the reaction mixture was recorded three times at 517 nm (for the DPPH assay), 734 nm (for the ABTS assay), and 593 nm (for the FRAP assay) using a Synergy HT microplate reader (BioTek Instruments, Winooski, VT, USA). The antioxidant activity was expressed as mg TE/g dry sample, with Trolox used as the standard antioxidant.

#### 3.10.2. Antihypertensive Activity

The ACE inhibitory activity of the BSEs was determined following the ACE kit-WST manual (Dojindo Molecular Technologies, Inc., Rockville, MD, USA). A 1% sample solution was prepared by dissolving the extract in HPLC-grade water. Then, 20 µL of the sample solution was added to a 96-well microplate together with 20 µL of deionized water for the blank1 and blank2 wells. Afterward, a substrate buffer (20 µL) was added to the sample and the blank1 and blank2 wells, while the blank2 wells had 20 µL of deionized water. The enzyme solution was added to the sample and blank2 wells and incubated at 37 °C for 1 h. Subsequently, 200 µL of the indicator working solution was added to each well and incubated at room temperature for 10 min. The microplate was then read at 450 nm using a microplate reader and captopril (0.1%) was used as a reference material. ACE inhibitory activity was calculated using the following formula and presented as a percentage (%):

(2) $$\mathrm{ACE}\;\mathrm{inhibitory}\;\mathrm{activity}(\%)=\frac{A_{blank1}-A_{sample}}{A_{blank1}-A_{blank2}}\times 100$$

where *A_blank_*_1_ is the absorbance of the positive control without ACE inhibition, *A_blank_*_2_ is the absorbance of the reagent blank, and *A_sample_* is the sample absorbance. 

#### 3.10.3. α-Glucosidase Inhibitory Activity

The inhibitory activity of the seaweed extracts against α-glucosidase from *Bacillus stearothermophilus* (70 U mg^−1^, Megazyme, Ireland) was measured following the method previously described with slight modifications [62]. Briefly, a sample solution (10 mg mL^−1^, 50 µL) was mixed with potassium phosphate buffer (200 mM, 50 µL, pH 6.8) and α-glucosidase (0.2 U mL^−1^, 50 µL) in a 96-well plate and pre-incubated at 37 ℃ for 10 min. After adding pNPG (3 mM, 100 μL), the reaction was allowed to continue for another 10 min. To stop the reaction, Na_2_CO_3_ (0.2 M, 750 μL) solution was added, and the absorbance was measured at a wavelength of 405 nm, using acarbose (0.1%) as a reference material. The reagents and samples were dissolved in potassium phosphate buffer (200 mM, pH 6.8). The samples and reaction systems without added enzymes were used as blank and background controls, respectively. The α-glucosidase inhibition rate of the samples was calculated using the following formula:

(3) $$\mathrm{Inhibition}\;\mathrm{rate}(\%)=\frac{A_{blank}-A_{sample}+A_{background}}{A_{blank}}\times 100$$

where *A_blank_*, *A_background_*, and *A_sample_* correspond to the absorbance of the blank control, the background control, and the sample, respectively.

### 3.11. Statistical Analysis

The data in this experiment were analyzed using SPSS software (version 27, IBM, Chicago, IL, USA). The results are expressed as the mean ± standard deviation (SD) (*n* = 3 replicates), and Duncan’s test was used for post hoc comparisons, at a significance level of *p* < 0.05. Correlations were investigated using Pearson’s correlation analysis.

## 4. Conclusions

In conclusion, this study explored the potential of SWE to prepare extracts from three important brown seaweed species collected from the coastal waters of South Korea. This research reveals that SWE can be a suitable green technique for valorizing seaweed and enhance their commercial application. The findings also show that the bioactive compounds can vary among the species. The BSEs showed high content of phlorotannin and other bioactive compounds. The obtained extracts proved to be a rich source of natural antioxidants. In addition, the extracts had antihypertensive and α-glucosidase inhibitory activity, which could be potential natural treatments for hypertension and diabetes. Moreover, the BSEs demonstrated antibacterial activity against foodborne pathogens, indicating that they could be a natural alternative to synthetic antimicrobial agents. Overall, this study highlights the potential of brown seaweed extracts as a source of natural bioactive compounds with various health benefits. These findings could be valuable for the development of functional foods, cosmetics, and pharmacologically active products. Further research is necessary to fully understand the mechanisms behind the biological activities of brown seaweed extracts and to explore their potential applications in the food, pharmaceutical, and cosmetic industries.

## Figures and Tables

**Table 1:** Extraction efficiency and color properties of BSEs obtained through subcritical water extraction.

Species	Extraction Efficiency (%)	Color			MRPs		
		L*	a*	b*	294 nm	420 nm	294/420
*S. thunbergii*	68.40 ± 0.44 ^b^	32.23 ± 0.26 ^ab^	1.63 ± 0.10 ^b^	0.60 ± 0.18 ^b^	2.851 ± 0.017 ^b^	0.306 ± 0.001 ^ab^	9.328 ± 0.029 ^b^
*U. pinnatifida*	81.88 ± 1.35 ^a^	34.42 ± 0.89 ^a^	4.53 ± 0.38 ^a^	2.92 ± 0.33 ^a^	2.163 ± 0.028 ^c^	0.188 ± 0.003 ^c^	11.506 ± 0.155 ^a^
*S. japonica*	69.02 ± 0.92 ^b^	30.33 ± 2.34 ^b^	1.63 ± 0.23 ^b^	0.48 ± 0.22 ^b^	2.980 ± 0.029 ^a^	0.305 ± 0.008 ^ab^	9.772 ± 0.156 ^b^

Values are expressed as the mean ± SD. Different letters indicate significant differences (*p* < 0.05) according to Duncan’s multiple range test.

**Table 2:** Chemical properties of BSEs from different brown seaweed species.

Species	Chemical Properties		
	Total Phlorotannin (mg PGE/g of Dry Sample)	Total Sugar (mg glucose/g of Dry Sample)	Reducing Sugar (mg glucose/g of Dry Sample)
*S. thunbergii*	38.82 ± 0.17 ^a^	116.66 ± 0.19 ^a^	53.27 ± 1.57 ^a^
*U. pinnatifida*	31.32 ± 0.50 ^b^	23.88 ± 0.42 ^c^	39.03 ± 0.52 ^c^
*S. japonica*	37.94 ± 0.30 ^a^	33.27 ± 0.51 ^b^	46.00 ± 1.82 ^b^

The values are expressed as the mean ± SD. Different letters indicate significant differences (*p* < 0.05) according to Duncan’s multiple range test.

**Table 3:** Monosaccharide compounds and molecular weight analysis of BSEs from different brown seaweed species.

Species	Monosaccharides Composition (%)					Molecular Weight (Da)		
	Fucose	Galactose	Glucose	Xylose	Mannose	Mn	Mw	PI
*S. thunbergii*	36.71	21.49	22.96	11.37	7.47	498	1514	3.04
*U. pinnatifida*	19.55	48.84	17.99	4.02	9.61	444	1145	2.58
*S. japonica*	29.24	46.64	14.39	4.99	4.73	331	982	2.97

**Table 4:** Biological activity of BSEs from different brown seaweed species.

Species	Antioxidant Activities			Antihypertensive Activity (%)	α-Glucosidase Inhibitory Activity (%)
	ABTS^+^ Radical Scavenging (mg TE/g of Dry Sample)	DPPH Radical Scavenging (mg TE/g of Dry Sample)	FRAP Assays (mg TE/g of Dry Sample)		
*S. thunbergii*	88.62 ± 2.35 ^b^	45.56 ± 0.23 ^a^	34.47 ± 0.49 ^a^	56.55 ± 0.26 ^c^	68.05 ± 1.15 ^b^
*U. pinnatifida*	79.13 ± 1.78 ^c^	35.29 ± 0.24 ^b^	27.02 ± 0.56 ^b^	58.71 ± 0.10 ^b^	38.52 ± 0.80 ^c^
*S. japonica*	124.77 ± 2.47 ^a^	46.35 ± 0.01 ^a^	33.95 ± 0.51 ^a^	59.77 ± 0.14 ^b^	34.74 ± 1.49 ^d^
Reference material	-			99.60 ± 0.28 ^a^	99.28 ± 0.26 ^a^

The values are expressed as the mean ± SD. Different letters indicate significant differences (*p* < 0.05) according to Duncan’s multiple range test. The reference material for antihypertensive activity is captopril (0.1%) and for α-glucosidase inhibitory activity, it is acarbose (0.1%).

**Table 6:** In vitro antimicrobial (well diffusion method) of BSEs against foodborne pathogens.

Conditions (mg/mL)		Gram-Positive		Gram-Negative	
		*B. cereus*	*S. aureus*	*E. coli*	*S. enterica*
		Inhibition Zone (mm)			
*S. thunbergii*	10	-	13	-	-
	20	14	17	11	-
	30	15	18	13	-
	40	17	19	14	12
	50	18	22	14	14
*U. pinnatifida*	10	-	-	-	-
	20	-	-	-	-
	30	-	11	-	-
	40	11	14	-	-
	50	13	15	11	-
*S. japonica*	10	-	12	-	-
	20	-	15	-	-
	30	-	16	-	-
	40	12	17	-	13
	50	14	18	-	14

## Data Availability

The original data presented in the study are included in the article/Supplementary Material; further inquiries can be directed to the corresponding author. The data presented in this study are available on request from the corresponding author.

## Supplementary Materials

The following supporting information can be downloaded at: https://www.mdpi.com/article/10.3390/md21060328/s1. Figure S1: Visual appearance of 3 types of brown seaweeds extracts obtained using subcritical water; Figure S2: Monosaccharides composition chromatograms of BSEs; Figure S3: GPC Chromatogram of BSE; Table S1: Proximate composition (%) of three brown seaweeds used in this experiment: Table S2: Pearson’s correlation coefficients of MRPs and sugar contents.
