# Supplementary material for: Exploring Bioactive Compounds in Brown Seaweeds Using Subcritical Water: A Comprehensive Analysis
Source: Mar Drugs. 2023 May 26;21(6):328. doi: 10.3390/md21060328 (PMC10303104; doi:10.3390/md21060328)
Supplement: Supplementary file 1 [file marinedrugs-21-00328-s001.zip › marinedrugs-2397445-supplementary.pdf]

**Exploring Bioactive Compounds in Brown Seaweeds Using Subcritical Water:**

**A Comprehensive Analysis**

Supplementary Data

**Table S1.** Proximate composition (%) of three brown seaweeds used in this experiment.

| Seaweed | Moisture | Ash | Crude Lipid | Crude Protein | Carbohydrate |
|---|---|---|---|---|---|
| ***S. thunbergii*** | 9.75±0.17$^{b}$ | 26.02±0.34$^{a}$ | 13.64±0.13$^{a}$ | 1.01±0.15$^{c}$ | 49.58±0.02$^{c}$ |
| ***U. pinnatifida*** | 5.33±1.33$^{bc}$ | 20.62±0.98$^{b}$ | 1.15±0.87$^{b}$ | 18.33±0.43$^{a}$ | 54.57±0.12$^{b}$ |
| ***S. japonica*** | 10.44±0.14$^{a}$ | 16.79±0.98$^{c}$ | 1.38±0.19$^{b}$ | 8.53±0.22$^{b}$ | 62.87±0.94$^{a}$ |

- Values are expressed as mean ± SD.
- Different letters indicate significant differences ($p$<0.05) according to Duncan’s multiple range test.

**Table S2.** Pearson's correlation coefficients of MRPs and sugar contents.

| Trait | a294 | a420 | a294/a420 | TSC | RSC |
|---|---|---|---|---|---|
| **a294** | 1 | 0.987** | -0.936** | 0.451ns | 0.761* |
| **a420** | - | 1 | -0.980** | 0.580ns | 0.841** |
| **a294/a420** | - | | 1 | -0.720* | -0.914** |

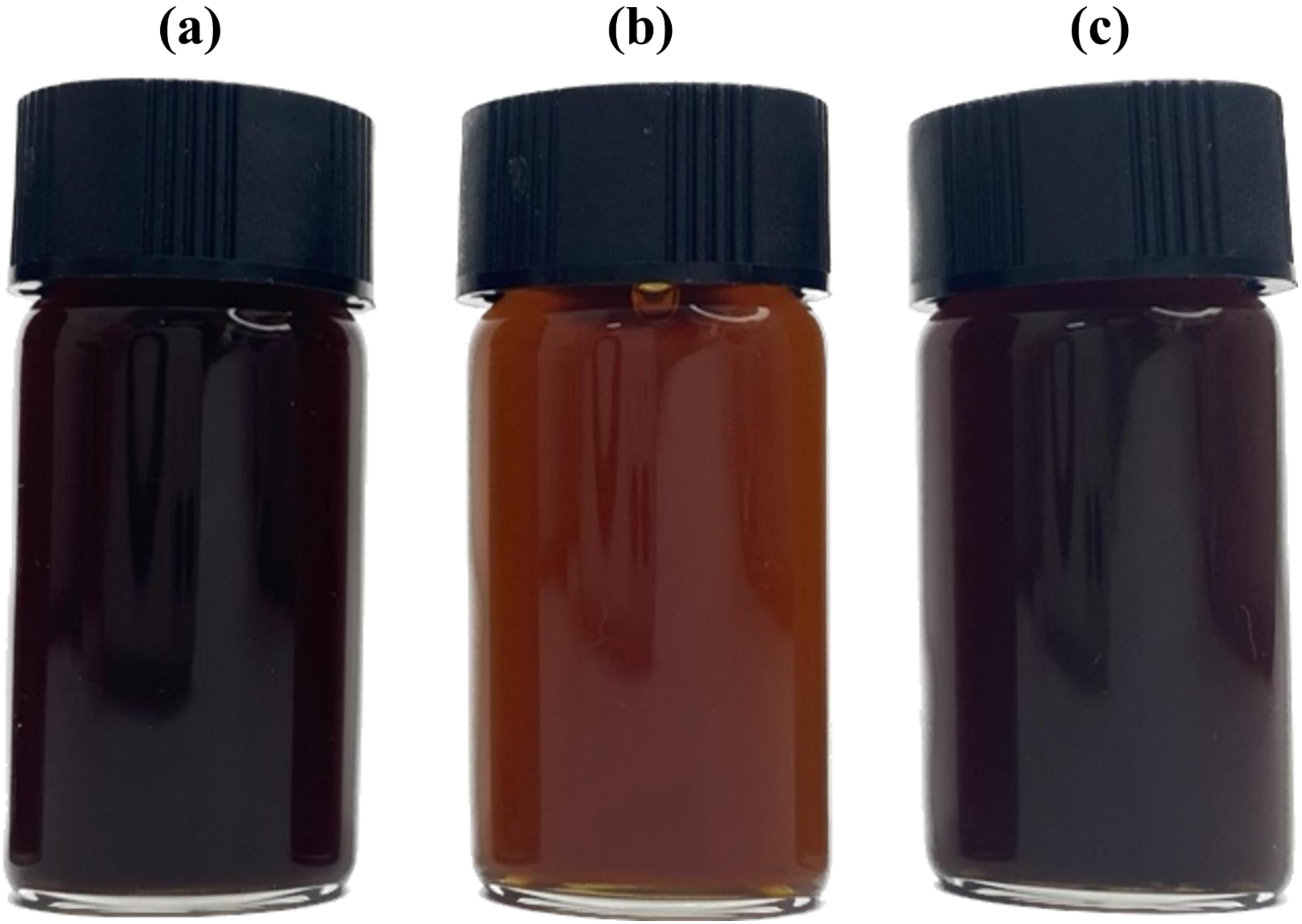


**Figure S1.** Visual appearance of 3 types of brown seaweeds extracts obtained using subcritical water ( a: *S. thunbergii*, b: *U. pinnatifida*, c: *S. japonica*) .

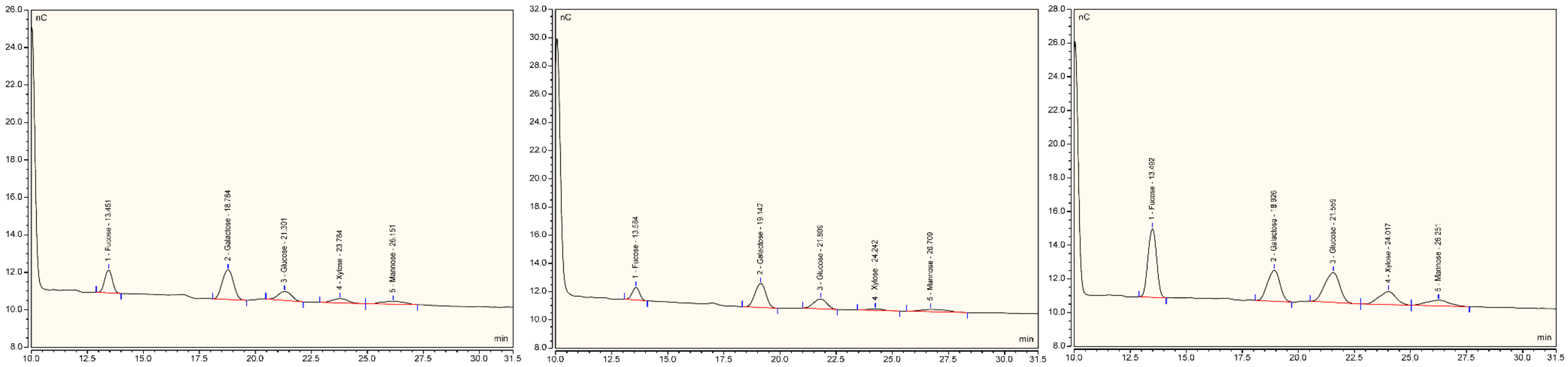


**Figure S2.** Monosaccharides composition chromatograms of BSEs.

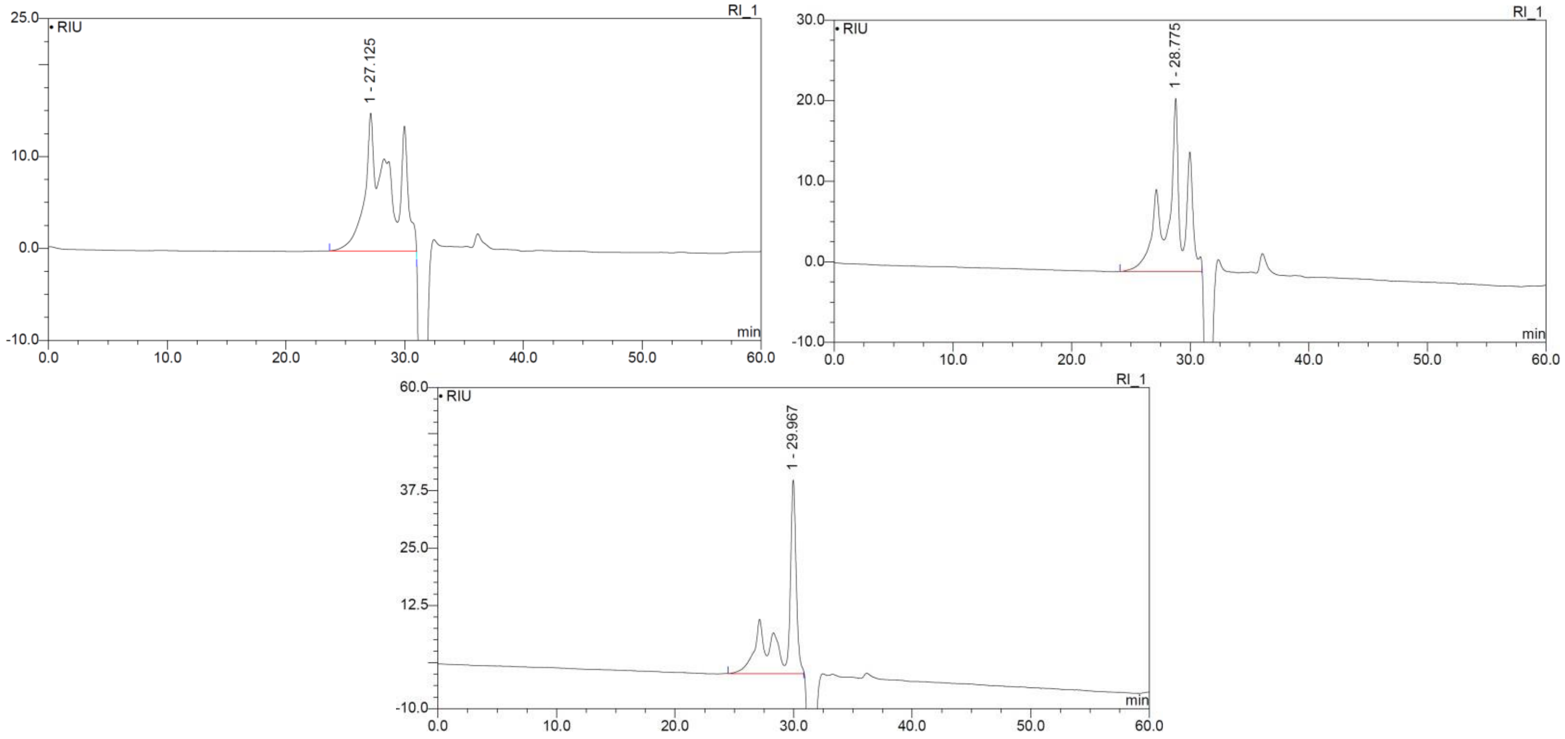


**Figure S3.** GPC Chromatogram of BSE.
